# Impacts of Endocrine Disruptor di-*n*-Butyl Phthalate Ester on Microalga *Chlorella vulgaris* Verified by Approaches of Proteomics and Gene Ontology

**DOI:** 10.3390/molecules25184304

**Published:** 2020-09-19

**Authors:** Chien-Sen Liao, Yong-Han Hong, Yoshikazu Nishikawa, Eriko Kage-Nakadai, Tai-Ying Chiou, Chien-Chang Wu

**Affiliations:** 1 Department of Biological Science and Technology, I Shou University, Kaohsiung 82445, Taiwan; 2 Graduate School of Human Life Science, Osaka City University, Osaka 558-8585, Japan; nisikawa@life.osaka-cu.ac.jp (Y.N.); nakadai@life.osaka-cu.ac.jp (E.K.-N.); 3 Department of Nutrition, I Shou University, Kaohsiung 84001, Taiwan; yonghan@isu.edu.tw; 4 Department of Nutrition and Food Sciences, Tezukayama Gakuin University, Osaka 590-0113, Japan; 5 School of Regional Innovation and Social Design Engineering, Kitami Institute of Technology, Hokkaido 090-8507, Japan; tkyuu@mail.kitami-it.ac.jp; 6 Institute of Biotechnology and Chemical Engineering, I-Shou University, Kaohsiung 84001, Taiwan; Fn802183@gmail.com

**Keywords:** *Chlorella vulgaris*, di-*n*-butyl phthalate, algal acute biotoxicity, proteomics, gene ontology

## Abstract

Di-*n*-butyl phthalate (DBP) is an extensively used plasticizer. Most investigations on DBP have been concentrated on its environmental distribution and toxicity to humans. However, information on the effects of plasticizers on algal species is scarce. This study verified the impacts of endocrine disruptor di-*n*-butyl phthalate ester on microalga *Chlorella vulgaris* by approaches of proteomics and gene ontology. The algal acute biotoxicity results showed that the 24h-EC50 of DBP for *C. vulgaris* was 4.95 mg L^−1^, which caused a decrease in the chlorophyll a content and an increase in the DBP concentration of *C. vulgaris*. Proteomic analysis led to the identification of 1257 *C. vulgaris* proteins. Sixty-one more proteins showed increased expression, compared to proteins with decreased expression. This result illustrates that exposure to DBP generally enhances protein expression in *C. vulgaris*. GO annotation showed that both acetolactate synthase (ALS) and GDP-L-fucose synthase 2 (GER2) decreased more than 1.5-fold after exposure to DBP. These effects could inhibit both the valine biosynthetic process and the nucleotide-sugar metabolic process in *C. vulgaris*. The results of this study demonstrate that DBP could inhibit growth and cause significant changes to the biosynthesis-relevant proteins in *C. vulgaris*.

## 1. Introduction

Microalgae are a large and diverse group of aquatic organisms which can be found in freshwater, estuarine, and marine environments [1,2]. Compared to vascular plants of the same footprint, microalgae are potential candidates for biofuel production due to their high photosynthetic efficiency, high biomass yield, fast growth rate, and high applicability [3]. Typically, the carbon content in microalgae exceeds 50%, which is higher than that in woods (around 40%), and their sulfur content is also low (less than 0.4%) [4]. In addition, microalgae have also attracted considerable interest as potential feedstock for bioactive natural chemicals; in particular, certain species under specific cultivation conditions can produce many useful materials, such as polysaccharides, carotenoids, oleic acid, and astaxanthin [5].

*Chlorella vulgaris* is a green unicellular microalga. For the earth’s carbon cycle, *C. vulgaris* is highly efficient in carbon capture and storage [6] and can regulate the carbon dioxide in the atmosphere [7]. *C. vulgaris* is a promising source of bioenergy and may also be a good alternative to current biofuel crops such as soybean, corn, or rapeseed, as it is more productive and does not compete with food production [8,9]. The artificial culture method of *C. vulgaris* can be divided into outdoor and indoor culture systems. The outdoor culture system usually uses the model of open-air or raceway-type ponds [10]. Photobioreactors (PBRs) and closed-column culture models are typically used for the indoor culture system [11,12]. However, irrespective of the culture methods and the process of cultivation, the culture system is easily exposed to environmental pollution, such as nanoparticles, chemical pollutants from seawater or groundwater, and the release of plastic tubing [13].

Endocrine-disrupting chemicals (EDCs) are defined as chemicals that have disruptive effects on the hormones of both wildlife and humans. Phthalate esters (PAEs), which are suspected to be EDCs, have been extensively used as plasticizers in various fields, such as food packaging, cosmetics, pesticides, building materials, chemical fertilizer, and the pharmaceutical industry [14,15,16,17,18]. As plasticizers, PAEs are characterized by their low water solubility and high octanol/water partition coefficients, but they are not covalently bound to plastics [19]. Due to their physicochemical properties and wide applications, PAEs are widely distributed in various environmental samples, such as groundwater [20], surface water [21], sediment [22], and soil [23]. Meanwhile, some PAEs are also semi-volatile organic compounds (SVOC) and can be present as indoor pollutants and easily leach into the air, car, air conditioner and household dust [24,25,26].

**Figure 1:**
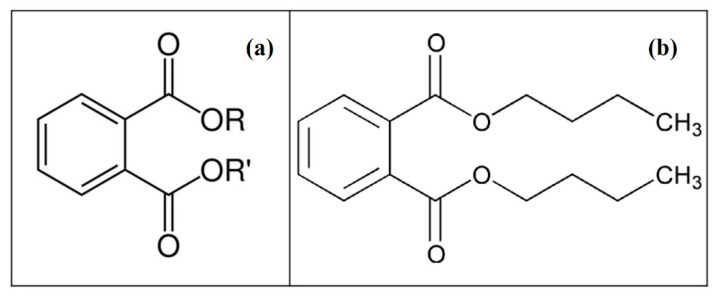
Chemical structure of (**a**) phthalate esters (PAEs) and (**b**) di-*n*-butyl phthalate (DBP).

Di-*n*-butyl phthalate (DBP) is one of the most widely used PAEs and is mainly used as a plasticizer for plastics. The chemical structure of PAEs and DBP are shown in Figure 1. It is an oily liquid that is soluble in fat and slightly soluble in water. It is not very volatile, so it does not readily vaporize into the atmosphere. It has a water solubility of 10 mg L^−1^ at 20 °C and a half-life of 22 years in aqueous solution [27]. In recent years, DBP has been reported to be found in surface water, sediments, drinking water [28], and even in human drugs [29]. According to the previous study, DBP was estimated to be 46.4% in drinking water, and the other three PAEs, namely dimethyl phthalate (DMP), diethyl phthalate (DEP), and di-2-ethylhexyl phthalate (DEHP), were 4.5%, 6.8%, and 39.4%, respectively [30]. In recent years, accumulation of DBP in aquatic environments and agricultural land has dramatically increased because of wastewater disposal and high agricultural products, such as fertilizers, pesticides, and the use of agricultural plastic mulch bags [31]. The DBP released into the environment can be taken up by crops and then enter the food chain. It can also be released into the algae artificial culture system through the same route and might potentially impact the physiology of algae and contaminate its end-products.

Several studies related to the algal biotoxicity of DBP have been reported. The effects of DBP on the growth rate and chlorophyll concentration were first reported in Reference [32]. This study revealed that the tolerance of the three microalgae species to DBP followed the order *Cylindrotheca closterium* > *Chaetoceros muelleri* > *Dunaliella salina*. Green alga (*D. salina*) was more sensitive to DBP, while benthic diatom (*C. closterium*) exhibited a strong adaptive ability to it. The other study investigated the photosynthetic performance of photosystem II (PSII), oxidative stress, and the antioxidant reaction induced by reactive oxygen species (ROS) in *C. vulgaris*, under the stress of cetyltrimethylammonium chloride (CTAC) [33]. However, only a few studies have reported the biotoxic effect of chemical pollutants on microalgae and without discussing the proteomics and gene ontology (GO) analysis.

Proteomics involves investigation of protein–protein interactions and the large-scale determination of gene and cellular function at the proteome level. Mass spectrometry (MS), such as liquid chromatography tandem mass spectrometry (LC–MS/MS), has increasingly become the method of choice for analysis of complex protein samples [34,35]. The analyzed LC–MS/MS data are uploaded onto a software database such as FunRich (http://www.funrich.org), to obtain the cellular GO annotations through computational comparison. GO is a major bioinformatics initiative to unify the representation of gene and gene product attributes across all species. There are three ontologies in GO, namely biological process (BP), cellular component (CC), and molecular function (MF). These three GO ontologies are disjoint, implying that there is no relation between the terms from the different ontologies. GO annotations consist of an association between a gene and a GO term, with supporting evidence in the form of a GO evidence code and either a published reference or description of the methodology used to create the annotation [36]. In order to investigate the effects of chemical pollutants on microalgae, a proteome and gene ontology comparison was performed. The purpose of this study was to verify the impacts of endocrine disruptor DBP on microalga *C. vulgaris* by approaches of proteomics and gene ontology. This included analyzing the acute biotoxicity of DBP on *C. vulgaris* and investigating the proteome and gene ontology of *C. vulgaris* exposed to DBP, using LC–MS/MS and related database software. The ultimate goal of this study is to verify more impacts information of endocrine disruptor DBP on *C. vulgaris* by approaches of proteomics and gene ontology.

## 2. Materials and Methods

### 2.1. Chemicals

The standard reagent, DBP (98.7% purity, CAS: 84-74-2), was purchased from Riedel-deHaën Co., Germany. Chlorophyll *a* standard (from spinach, CAS: 479618) was purchased from Sigma-Aldrich Chemical Co., MI, USA. The solvents used in this experiment, including ethanol and acetone, were purchased from E. Merck (Germany). All other chemicals were purchased from Sigma-Aldrich Chemical Co., MI, USA. All reagents used were reagent HPLC-grade. Individual DBP stock solutions were dissolved with acetone at 10,000 mg L^−1^ before use. The glassware was thoroughly cleaned to reduce any background contamination of PAEs. All glassware was washed with deionized water and dried at 80 °C, overnight, in an oven. After cooling, the glassware was rinsed twice with acetone and air-dried for use.

### 2.2. Cultivation of Microalgae

The freshwater unicellular green alga *C. vulgaris* Beij. (strain #3001) was chosen as the testing species in this study. *C. vulgaris* was obtained from the Biodiversity Research Center, Academia Sinica, Taipei, Taiwan. The initial incubation of *C. vulgaris* was carried out, using 60 L closed poly (methyl methacrylate) (PMMA) columns with an illumination of approximately 400 μmol·m^−2^·s^−1^ and maintained at 25 °C. The aeration provided air in flow rates of 30 L min^−1^ column^−1^. To maintain the log-growing cultures, the density of the cultures was maintained at the range of 2.5 to 2.8, at optical density (OD), at 680 nm, by diluting with medium every week. *C. vulgaris* was cultivated in the medium used in our previous study [37], and the pH of the medium was adjusted to 7 with potassium hydroxide, before autoclaving at 121 °C for 15 min.

### 2.3. Experimental Design

Algal acute biotoxicity assays were used to evaluate the DBP toxicity to *C. vulgaris*. Batch experiments were performed in the 300 mL bench glass-columns, and each column contained 250 mL of *C. vulgaris* culture medium. According to the growth curve of *C. vulgaris*, the biotoxicity assay started at the early log phase, which is the second or third day after adding fresh culture medium. Based on DBP found in the aquatic environment [38], DBP at concentrations of 1, 2, 3, 4, 5, 6, 7, 8, 9, and 10 mg L^−1^ were added to the culture medium. After 24 h, each 40 μL *C. vulgaris* sample solution was extracted with 960 μL absolute ethanol for 30 min, in the dark, and centrifuged at 1000× *g* for 10 min, to obtain chlorophyll *a*. Based on ISO 10,260 and the method of Arvola [39], the chlorophyll *a* content of *C. vulgaris* was spectrophotometrically quantified at an absorbance of 665 and 750 nm, respectively. The toxicity calculation was based on the content of chlorophyll *a* following the 24 h test period. The toxicity in a blank test without any DBP was also analyzed after the 24 h test period. The statistical calculation of these values was based on at least three replicates and generated error bars based on the standard error of the mean (SEM). The 24 h median effective concentration (24h-EC_50_) values for the inhibition of cell growth were evaluated to determine the DBP acute biotoxicity to *C. vulgaris*. The dose–response curves of DBP and EC_50_ followed the methods of Reference [40] and were calculated by an AAT Bioquest EC_50_ calculator (https://www.aatbio.com/tools/ec50-calculator). The *C. vulgaris* culture medium remaining after the acute biotoxicity assays was centrifuged at 8000× *g* for 10 min, and the supernatant was removed. The *C. vulgaris* samples were then precipitated and used for subsequent proteomics analysis.

### 2.4. Proteomics Analysis of C. vulgaris

#### 2.4.1. Protein Extraction and Gel Electrophoresis

Proteomics analysis was performed on normal *C. vulgaris* (control group, Con.) samples (*n* = 3) and DBP-exposed *C. vulgaris* (experimental group, Exp.) samples (*n* = 3). *C. vulgaris* sample precipitates were resuspended in 2.5 mL of Tris pH 8.8 buffered phenol and sonicated with a probe sonicator. The lysed samples were added to an equal volume of extraction buffer containing 10 mM EDTA, 0.4% 2-mercaptoethanol, 0.1 M Tris-HCl pH 8.8, and 0.9 M sucrose. The homogenate was mixed for 30 min and then centrifuged for 15 min, at 5000× *g*, 4 °C. The phenol phase was removed, and the proteins were precipitated with five volumes of ice-cold 0.1 M ammonium acetate in 100% methanol, overnight, at −20 °C. The homogenate was centrifuged for 10 min at 5000× *g*, 4 °C, and the protein pellet was transferred into 1.5 mL microfuge tubes after washing twice in 5 mL of ice-cold 0.1 M ammonium acetate in 100% methanol. Subsequently, the protein pellet was thoroughly washed twice in 1 mL of 80% ice-cold acetone with 10 mM DTT and a final wash in 1 mL of 70% Ethanol. Finally, the pellet was air-dried in a fume hood. The sample was analyzed by a 12.5% SDS-PAGE. After electrophoresis, the SDS-PAGE was stained with VisPRO Protein Stain Kit (Visual Protein, Taiwan) for 5 min. After being stained, the SDS-PAGE was washed in Milli-Q water and stored at 4 °C, until processing for in-gel digestion.

According to the method of Shevchenko, the SDS-PAGE gel lanes corresponding to the sample were cut in 2 slices, and each slice was processed for in-gel digestion. Briefly, slices were washed/rehydrated three times in 25 mM ABC (ammonium bicarbonate pH 7.9) + 50% ACN (acetonitrile)/50 mM ABC (ammonium bicarbonate pH 7.9). Subsequently, disulfide bonds were reduced with 10 mM DTT for 1 h at 56 °C and alkylated with 25 mM iodoacetamide for 45 min at 25 °C in the dark. After two subsequent wash cycles, the slices were dried and incubated overnight with 20 ng μL^−1^ MS-grade Trypsin Gold (Promega, Madison, WI). Peptides were extracted three times in 10 μL of 50% ACN in 1% formic acid. The volume was dried out in a vacuum centrifuge prior to LC–MS/MS analysis.

#### 2.4.2. Nano-LC Separation and Mass Spectrometry

Peptides were separated by using an Ultimate System 3000 Nano-LC system (Thermo Fisher Scientific, Bremen, Germany) equipped with a 75 μm inside diameter (ID) and 25 cm length C18 Acclaim PepMap Nano-LC column (Thermo Fisher Scientific, San Jose, CA, USA) and packed with 2 μm particles with a pore size of 100 Å. Mobile phase A was 0.1% formic acid in water, and mobile phase B was 100% acetonitrile with 0.1% formic acid. A segmented gradient from 2% to 35% solvent B for 90 min, at a flow rate of 300 mL/min, and a column temperature of 35 °C were used. Intact peptide mass spectra and fragmentation spectra were acquired on a Thermo Scientific ™ Orbitrap Fusion ™ Lumos ™ Tribrid ™ Mass Spectrometer (Thermo Fisher Scientific, UK). Mass spectrometry analysis was performed in a data-dependent mode with Full-MS (externally calibrated to a mass accuracy of <5 ppm, and a resolution of 120,000 at *m*/*z* = 200), followed by high-energy collision activated dissociation (HCD)-MS/MS of the most intense ions in 3 s. HCD-MS/MS (resolution of 15,000) was used to fragment multiply charged ions (charge state 2-7) within a 1.4 Da isolation window at a normalized collision energy of 32 eV. Automatic gain control (AGC) target at 5e^5^ and 5e^4^ was set for MS and MS/MS analysis, respectively, with previously selected ions dynamically excluded for 180 s.

#### 2.4.3. Label-Free Peptide Quantification and Identification

The raw peptide mass spectra were loaded onto the proteome discoverer for proteomics software (v2.2, Thermo Fisher, Hemel Hempstead, UK). The spectra were used to validate protein identification, using MASCOT (www.matrixscience.com). All MS/MS spectra were exported from proteome discoverer software searched against genus *Chlorella* (20,810 sequences; 11,425,167 residues). The search parameters used were as follows: peptide mass tolerance of 20 ppm and MS/MS mass tolerance of 0.02 Da; up to two missed cleavages, cysteine carbamidomethylation set as the fixed modification, and methionine oxidation, asparagine deamidation, glutamine deamidation, and *N*-terminal acetylation set as variable modifications. A decoy database search was performed to determine the peptide false discovery rate (FDR) with the Target Decoy PSM Validator module. A 0.05% peptide FDR threshold was applied. Additionally, all protein identifications had a MASCOT score >25 and protein FDR confidence high level. Then protein quantities were calculated by unique peptide intensity and normalization by total peptide amount.

### 2.5. Proteomics and Gene Ontology Analysis

The Uniprot database was used for proteomics and gene ontology analysis. The taxonomy of *Chlorella* genus was selected and used as the database for the comparison of the LC–MS/MS data (https://www.uniprot.org/taxonomy/3071). Then, the LC–MS/MS data were uploaded from a Microsoft Excel spreadsheet onto FunRich software (http://www.funrich.org). FunRich (v3.1.3) generates gene ontology analysis to describe BP, CC, or MF between control and experimental groups. Statistical analyses of three datasets were performed by unpaired Student’s *t*-test. All results were expressed as means with their standard deviation (SD). A *p*-value <0.05 was taken as the minimum level of significance.

## 3. Results

### 3.1. Algal Acute Biotoxicity Assays

**Figure 2:**
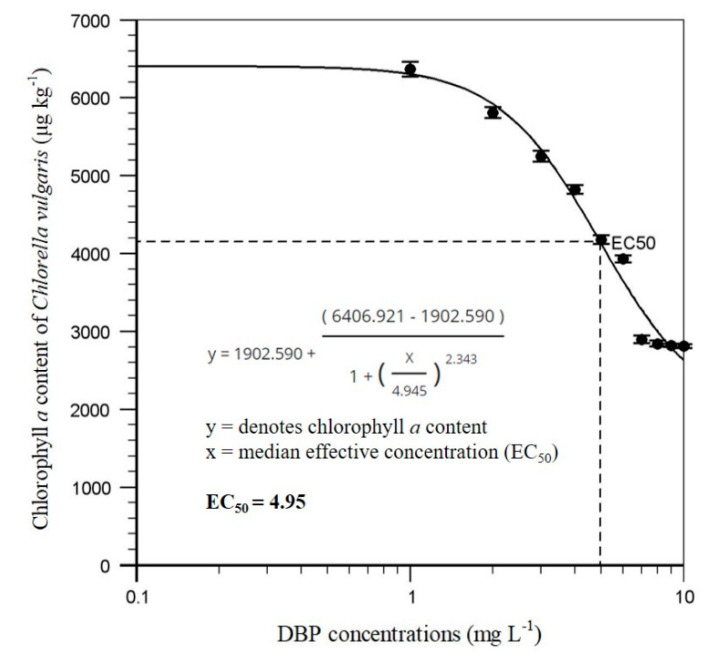
Dose–response curve of algal acute biotoxicity, and 24h-EC_50_ of DBP for *C. vulgaris*. Data from three measurements are presented as mean ± SE.

The chlorophyll *a* content of *C. vulgaris* after exposure to different concentrations of DBP is shown in Figure 2. The result indicates that the 24h-EC_50_ of DBP for *C. vulgaris* was 4.95 mg L^−1^. The control test referred to acute biotoxicity analysis without DBP treatment. The result shows that the chlorophyll *a* content of *C. vulgaris* decreased with the increase in DBP exposure concentration. This result also showed a slight growth inhibition of *C. vulgaris* after DBP exposure. Our previous report demonstrated that the acute biotoxicity of DBP to *C. vulgaris* after 24 h of exposure could decrease the chlorophyll *a* content [41]. Another study indicated that DBP caused growth inhibition and the 96h-EC_50_ of DBP on two typical freshwater algae (*Scenedesmus obliquus* and *Chlorella pyrenoidosa*) was 15.3 and 3.14 mg L^−1^, respectively **[42]**. The other research study demonstrated that DBP impacted growth rate and chlorophyll concentration in three algae **[32]**. This study revealed that tolerance of the three microalgae species to DBP was in the order of *Cylindrotheca closterium* > *Chaetoceros muelleri* > *Dunaliella salina*. Green alga (*D. salina*) was more sensitive to DBP probably because it does not have a rigid cell wall composed of cellulose or of any other inert material. The cell coat of *D. salina* is primarily composed of glycoprotein with some neuraminic acid residues [43]. The latest research study revealed that the 96 h-IC_50_ value of DBP for *Chlorella pyrenoidosa* was 2.41 mg L^−1^ [44]. In order to clarify the biotoxicity of DBP at the genomic and proteomic level, a proteomics analysis of *C. vulgaris* was required.

### 3.2. Proteome Expression Analysis

Table 1 shows the summary of protein quantifications in *C. vulg*aris. The results show that the amount of protein was sufficient for subsequent analysis. The protein amounts of Con. and Exp. were both higher than the detection limit (60 μg). The expression results of these two test groups are shown in Table 2. The results revealed that a total of 1257 *C. vulgaris* proteins were identified in the DBP exposed sample. Among these proteins, the expression of 659 proteins were increased, and 598 were decreased after DBP exposure. This result illustrated that DBP exposure generally induced protein expression in *C. vulgaris*, since 61 more proteins had increased expression, compared to the number of proteins with decreased expression. With the increase in protein expression level, the physiological responses might be more significant. Therefore, this study screened proteins with increase in expression by more than 1.5-fold, for subsequent proteome identification and GO annotation analysis. Further analysis revealed that the expression of 31 proteins was increased by more than 1.5 folds and the expression of 37 proteins was decreased by more than 1.5-fold, and these proteins were called differentially expressed proteins (DEPs) (Table 2).

### 3.3. Proteome Identification and GO Annotation and Comparison

**Figure 3:**
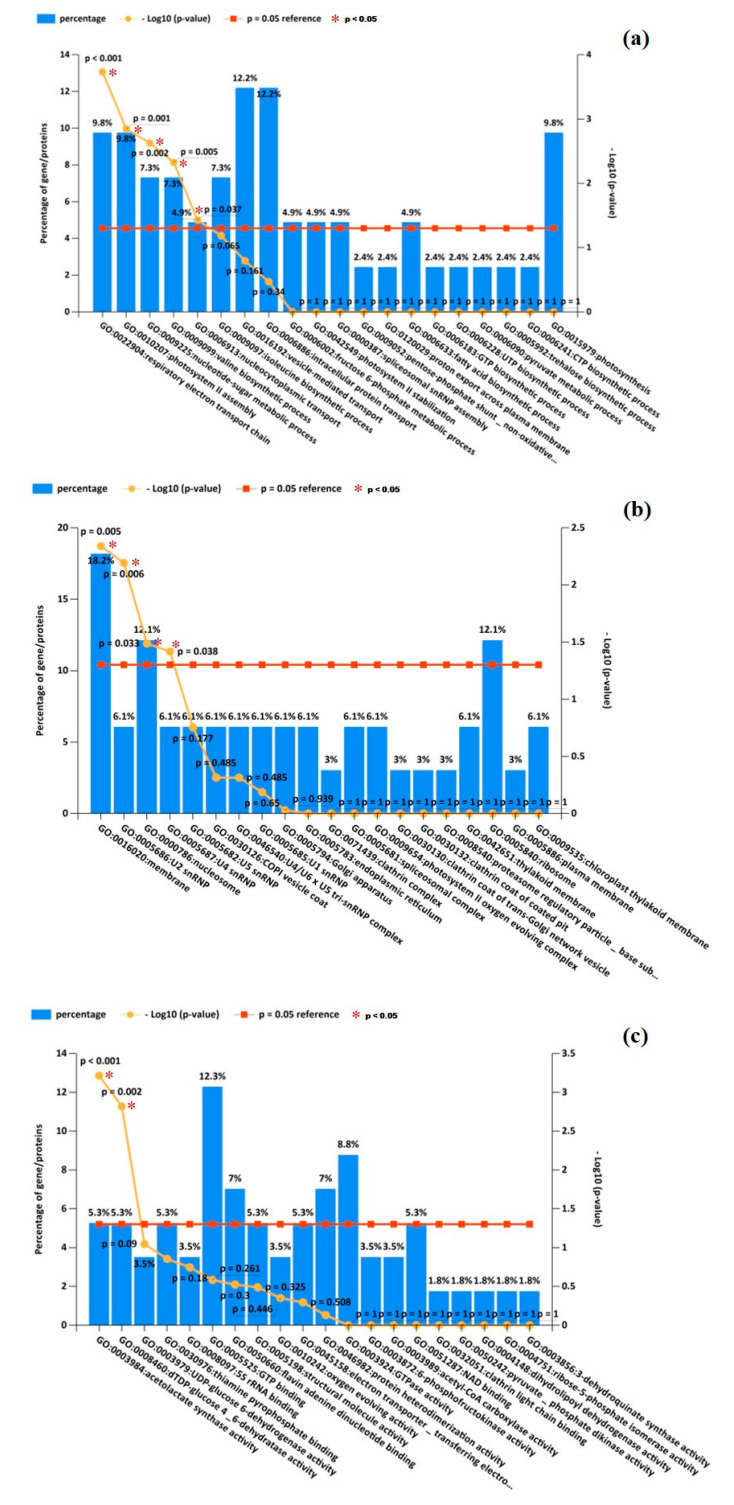
Top 20 gene ontology (GO) annotations of (**a**) biological process, (**b**) cellular component, and (**c**) molecular function cluster between Exp. and Con. The comparison arranged according to decreasing −log10 (*p*-value) (yellow line). Percentages of GO annotations belonging to each process are shown blue bars, −log10 (*p* = 0.05) is shown as a reference (red line). * = *p* < 0.05.

A total of 68 DEPs were used for database search for gene ontology analysis, which found that a total of 62 DEPs were successfully paired to the GO annotations. Forty-one DEPs were identified to be important for BP, 33 DEPs as CC, and 57 DEPs were important for MF. However, six DEPs could not be paired to any GO annotation. Figure 3 shows the top 20 GO annotations of BP, CC, and MF clusters between the Exp. and Con groups. In the figure, the comparison is arranged according to decreasing −log10 (*p*-value) (yellow line). Higher −log10 (*p*-value) implies higher statistical reliability of this analysis. The percentage of GO annotations belonging to each process is shown as blue bars, with −log10 (*p* = 0.05) being the reference of minimum threshold for statistical reliability (red line).

#### 3.3.1. Biological Process (BP)

The definition of the BP cluster is operations or sets of molecular events pertinent to the functioning of integrated living units, such as cells, tissues, organs, and organisms. Figure 3a shows the top 20 GO annotations of the BP cluster. All results were paired from the BP database of *Chlorella* genus. The result shows that the first GO annotation (GO:0022904: respiratory electron transport chain) has the highest statistical reliability. There were nine proteins identified in this data in the GO annotation database, and four out of the total 41 DEPs were paired with the BP. Therefore, the statistical ratio of effective pairing for this GO annotation is 9.8% (4/41). In Figure 3a, for four other GO annotations with higher statistical reliability than the reference value, the name and statistical ratio of effective pairings are GO:0010207: photosystem II assembly (4/41 = 9.8%), GO:0009225: nucleotide-sugar metabolic process (3/41 = 7.3%), GO:0009099: valine biosynthetic process (3/41 = 7.3%), and GO:0006913: nucleocytoplasmic transport (2/41 = 4.9%), respectively. In addition, although the statistical ratio of effective pairings for GO:0016192: vesicle-mediated transport and GO:0006886: intracellular protein transport are both 12.2% (5/41), the identified DEPs only account for 6.2% (5/81) and 5.3% (5/95) of these two GO annotations, with the reliability being lower than the reference. The data, therefore, were not considered.

#### 3.3.2. Cellular Component (CC)

The definition of the CC cluster is the parts of a cell or its extracellular environment. Figure 3b shows the top 20 GO annotations of the CC cluster. All results were paired from the CC database of *Chlorella* genus. The result shows that the first GO annotation (GO: 0016020: membrane) has the highest statistical reliability. There were 121 identified proteins in this GO annotation database, and six of total 33 DEPs were paired with the BP. Therefore, the statistical ratio of effective pairing for this GO annotation is 18.2% (6/33). In Figure 3b, three other GO annotations with higher statistical reliability than the reference value, the name, and statistical ratio of effective pairings are GO: 0005686: U2 snRNP (2/33 = 6.1%), GO: 0000786: nucleosome (4/33 = 12.1%), and GO: 0005687: U4 snRNP (2/33 = 6.1%), respectively. In addition, although the statistical ratio of effective pairing for GO:0005840: ribosome is 12.1% (4/33), the four identified DEPs only account for 1.2% (4/335) of this GO annotation, with the reliability being lower than the reference. The data, therefore, were not considered.

#### 3.3.3. Molecular Function (MF)

The definition of MF cluster is the elemental activities of a gene product at the molecular level, such as binding or catalysis. Figure 3c shows the top 20 GO annotations of the MF cluster. All results were paired with the MF database of *Chlorella* genus. The result shows that the first GO annotation (GO:0003984: acetolactate synthase activity) has the highest statistical reliability. There were four identified proteins in this GO annotation database, and three of total 57 DEPs were paired to the BP. Therefore, the statistical ratio of effective pairing for this GO annotation is 5.3% (3/57). Figure 3c shows another GO annotation with higher statistical reliability than the reference value, with its name and statistical ratio of effective pairing being GO:0008460: dTDP-glucose 4,6-dehydratase activity (3/57 = 5.3%). In addition, although the statistical ratio of effective pairing for GO:0005525: GTP binding is 12.3% (7/57), the seven identified DEPs only account for 2.9% (7/238) of this GO annotation, the reliability is lower than the reference. The data, therefore, were not considered.

## 4. Discussion

The algal acute biotoxicity assay results showed that the 24h-EC_50_ of DBP for *C. vulgaris* was 4.95 mg L^−1^, which resulted in a slight growth inhibition of *C. vulgaris* with increased exposure to DBP. In order to clarify the biotoxicity of DBP at the genome and proteome levels, proteomics analysis and GO annotation were performed. A total of 1257 *C. vulgaris* proteins were identified after exposure to DBP, among which 659 proteins exhibited increased expression, while the other 598 proteins exhibited decreased expression. This illustrates that exposure to DBP generally enhances protein expression in *C. vulgaris*.

Table 3 shows the identification and GO annotation results of DBPs in *C. vulgaris* from the Con. and Exp. According to the results, the DBP biotoxicity on *C. vulgaris* affects two major pathways at the proteome level: (1) valine biosynthetic process and (2) nucleotide-sugar metabolic process. Table 2 shows that the expression of a protein related to acetyl-CoA biosynthesis decreased 1.66~2.37-fold after DBP exposure. This protein is acetolactate synthase (ALS), also known as acetohydroxy acid synthase (AHAS), and it is widely found in plants, algae, and microorganisms. ALS is an enzyme specifically involved in the conversion of pyruvate to acetolactate. A previous study indicated that ALS uses thiamine pyrophosphate to link two pyruvate molecules to form acetolactate [45]. Acetolactate is the precursor of the three branched-chain amino acids—valine, leucine, and isoleucine—which are also essential amino acids for plants and algae. The GO annotation of *C. vulgaris* revealed that ALS activity (MF) and valine biosynthetic process (BP) were both decreased after DBP exposure. These results lead to the conclusion that DBP affected the ALS activity of *C. vulgaris*, inhibited the biosynthesis of valine, and finally caused the growth inhibition of *C. vulgaris*. This finding suggests that DBP has herbicide-like properties that can inhibit the growth of algae by reducing the biosynthesis of valine through ALS inhibition [46,47].

On the other hand, Table 2 also shows that the expression of GDP-L-fucose synthase 2 (GER2) decreased 2.82-fold after exposure to DBP. GDP-L-fucose was first discovered in 1958 [48], and its biosynthesis was found to be a common process in many life forms, such as bacteria, algae, plants, invertebrates, and vertebrates. GDP-L-fucose synthase (GER) is an enzyme that participates in the pathway of GDP-L-fucose biosynthesis. A previous study indicated that GER2 is involved in step two of the pathway that biosynthesizes GDP-L-fucose from GDP-alpha-D-mannose [49]. Additionally, the GDP-L-fucose biosynthesis pathway is also part of nucleotide-sugar biosynthesis. The GO annotation of *C. vulgaris* revealed that dTDP-glucose 4,6-dehydratase activity (MF) and nucleotide-sugar metabolic process (BP) were both decreased after DBP exposure. dTDP-glucose 4,6-dehydratase (RmlB) is an enzyme that participates in the dehydration pathway of dTDP-glucose. Just like GER2, it also participates in the biosynthesis of nucleotide-sugars. These results lead to the conclusion that DBP affected the GER2 of *C. vulgaris*, inhibited the nucleotide-sugar metabolic process, and caused the growth inhibition of *C. vulgaris*. It can be seen from these results that exposure of *C. vulgaris* to DBP inhibits enzymes related to the essential amino acids and nucleotide-sugar metabolic pathways in *C. vulgaris* and further inhibits the growth of *C. vulgaris*.

## 5. Conclusions

In summary, exposure to DBP could potentially affect the physiology of *C. vulgaris*. In algal acute biotoxicity, the 24h-EC_50_ of DBP for *C. vulgaris* was 4.95 mg L^−1^ and caused a decrease in the chlorophyll *a* content of *C. vulgaris* with increased exposure to DBP. After proteomics analysis, a total of 1257 *C. vulgaris* proteins were identified. Among them, 659 proteins had an increased expression, while 598 exhibited a decreased expression after exposure to DBP. This result illustrated that exposure of *C. vulgaris* to DBP generally reduced protein expression in *C. vulgaris*. Protein identification and GO annotation showed that both ALS and GER2 were decreased more than 1.5-fold after exposure to DBP. These effects could inhibit the valine biosynthetic process and nucleotide-sugar metabolic process in *C. vulgaris*. In summary, results from this study show that DBP could cause growth inhibition and significant decreases in the biosynthesis-relevant proteins in *C. vulg*aris. This study provided more impacts information of endocrine disruptor DBP on *C. vulgaris* by approaches of proteomics and gene ontology. This verify study could serve as a basis for further study and assist in developing strategies for avoiding such effects in the future.

## Figures and Tables

**Table 1:** Summary of protein quantifications in *C. vulgaris*.

Sample	Concentration (μg μL^−1^)	Amount (μg) ^a^
Con.1	9.97	997
Con.2	13.09	5236
Con.3	15.74	6296
Exp.1	17.4	1740
Exp.2	21.93	8772
Exp.3	19.30	7720

^a^ Protein amount > 60 μg. Exp. = experimental group; Con. = control group.

**Table 2:** Summary of expressed proteins in *C. vulgaris*.

	Expressed Proteins		Differential Expressed Proteins ^a^	
Exp. vs. Con.	Upregulation	Downregulation	Upregulation	Downregulation
	659	598	31	37

^a^ Fold change ≧ 1.5 or ≦ −1.5, *p*-value < 0.05.

**Table 3:** Identification and GO annotation results of diethyl phthalates (DEPs) in *C. vulgaris* from Exp. and Con.

Protein-ID	Protein Name	Exp. Group Average ^a^	Con. Group Average ^a^	Exp. vs. Con.	Biological Process	Cellular Component	Molecular Function
A0A2P6TB75	Small nuclear ribonucleoprotein E	2,360,960.93	690,303.1496	3.42		U2 snRNP (GO:0005686) U4 snRNP (GO:0005687)	
E1ZJG0	Small nuclear ribonucleoprotein E	2,360,960.93	690,303.1496	3.42		U2 snRNP (GO:0005686) U4 snRNP (GO:0005687)	
E1Z2X3	Histone H2A	1,447,145,818	528,512,569.9	2.74		nucleosome (GO:0000786)	
E1ZGZ5	Histone H2A	1,447,145,818	528,512,569.9	2.74		nucleosome (GO:0000786)	
E1Z345	Histone H2A	1,763,428,632	695,597,118.3	2.54		nucleosome (GO:0000786)	
A0A2P6U370	Putative histone H2A variant 3	1,763,428,632	695,597,118.3	2.54		nucleosome (GO:0000786)	
A0A2P6TN97	Chloroplast ATP synthase subunit delta	94,378,441.93	44,558,769.92	2.12		membrane (GO:0016020)	
E1ZUG4	Uncharacterized protein	94,378,441.93	44,558,769.92	2.12		membrane (GO:0016020)	
A0A2P6TDB7	Dihydrolipoyl dehydrogenase	188,274,201.3	95,700,812.66	1.97	photosystem II assembly (GO:0010207)		
E1ZQR2	Uncharacterized protein	187,902,521.2	95,700,812.66	1.96	photosystem II assembly (GO:0010207)		
E1ZA81	Uncharacterized protein	23,294,602.12	12,323,985.51	1.89	photosystem II assembly (GO:0010207)		
A0A2P6TVA7	THYLAKOID chloroplastic	23,294,602.12	12,323,985.51	1.89	photosystem II assembly (GO:0010207)		
A0A2P6TYV5	Prohibitin-mitochondrial-like	26,879,102.59	14,485,945.79	1.86		membrane (GO:0016020)	
A0A2P6U113	GTP-binding nuclear Ran	174,458,781	116,477,439.7	1.5	nucleocytoplasmic transport (GO:0006913)		
E1ZJ35	GTP-binding nuclear protein	174458781	116,477,439.7	1.5	nucleocytoplasmic transport (GO:0006913)		
E1ZSU0	Acetolactate synthase	13,983,262	23,251,122	−1.66	valine biosynthetic process (GO:0009099)		acetolactate synthase activity (GO:0003984)
A0A2P6TWL0	Guanylate-binding 4-like	1,129,475.663	2,130,899.706	−1.89		membrane (GO:0016020)	
E1Z5A9	Uncharacterized protein	1,129,475.663	2,130,899.706	−1.89		membrane (GO:0016020)	
A0A2P6TBH4	Acetolactate synthase	4,860,187.934	11,528,724.53	−2.37	valine biosynthetic process (GO:0009099)		acetolactate synthase activity (GO:0003984)
A0A2P6TBH6	Acetolactate synthase	4,860,187.934	11,528,724.53	−2.37	valine biosynthetic process (GO:0009099)		acetolactate synthase activity (GO:0003984)
F2YGL6	Cytochrome b6	6,172,062.4	15,644,836.86	−2.53	respiratory electron transport chain (GO:0022904)		
A0A2I4S6M4	Cytochrome b6	6,172,062.4	15,644,836.86	−2.53	respiratory electron transport chain (GO:0022904)		
C0KRE8	Cytochrome b6 (Fragment)	6,172,062.4	15,644,836.86	−2.53	respiratory electron transport chain (GO:0022904)		
A0A2P6U4I5	GDP-L-fucose synthase 2	954,277.2115	2,692,354.388	−2.82	nucleotide-sugar metabolic process (GO:0009225)		dTDP-glucose 4, 6-dehydratase activity (GO:0008460)
E1Z7Y1	Uncharacterized protein	954,277.2115	2,692,354.388	−2.82	nucleotide-sugar metabolic process (GO:0009225)		dTDP-glucose 4, 6-dehydratase activity (GO:0008460)
A0A2P6TR93	GDP-L-fucose synthase 2	954,277.2115	2,692,354.388	−2.82	nucleotide-sugar metabolic process (GO:0009225)		dTDP-glucose 4, 6-dehydratase activity (GO:0008460)
P56321	Cytochrome b6	3,652,260.183	11,782,273.75	−3.23	Respiratory electron transport chain (GO:0022904)		

^a^ Fold change ≧ 1.5 or ≦ −1.5, *p*-value < 0.05.
